# Combined Use of Clips and Nylon Snare (“Tulip-Bundle”) as a Rescue Endoscopic Bleeding Control in a Mallory-Weiss Syndrome

**DOI:** 10.1155/2014/972765

**Published:** 2014-09-25

**Authors:** Hrvoje Ivekovic, Bojana Radulovic, Suzana Jankovic, Pave Markos, Nadan Rustemovic

**Affiliations:** Department of Gastroenterology and Hepatology, Hospital Centre Zagreb, Kispaticeva 12, 10000 Zagreb, Croatia

## Abstract

Mallory-Weiss syndrome (MWS) accounts for 6–14% of all cases of upper gastrointestinal bleeding. Prognosis of patients with MWS is generally good, with a benign course and rare recurrence of bleeding. However, no strict recommendations exist in regard to the mode of action after a failure of primary endoscopic hemostasis. We report a case of an 83-year-old male with MWS and rebleeding after the initial endoscopic treatment with epinephrine and clips. The final endoscopic control of bleeding was achieved by a combined application of clips and a nylon snare in a “tulip-bundle” fashion. The patient had an uneventful postprocedural clinical course and was discharged from the hospital five days later. To the best of our knowledge, this is the first case report showing the “tulip-bundle” technique as a rescue endoscopic bleeding control in the esophagus.

## 1. Introduction

Mallory-Weiss syndrome (MWS) refers to a bleeding from vomiting-induced tear of the mucosa at the gastroesophageal junction or gastric cardia. The MWS causes approximately 6–14% of all causes of upper gastrointestinal bleeding [1]. Risk factors for the MWS include chronic alcohol consumption, aspirin use, and episodes of increased intra-abdominal pressure such as paroxysms of coughing, pregnancy, heavy lifting, straining, seizure, blunt abdominal trauma, colonic lavage, and cardiopulmonary resuscitation [2]. Moreover, the MWS is well-known complication of upper endoscopy, with the reported prevalence of 0,07–0,45% [3]. Although the majority of patients have a benign course of disease, in those with a high-risk stigmata due to advanced age, low hemoglobin level, severe comorbidity, a fatal outcome may occur [4].

In patients with the MWS and active bleeding or exposed vessels, the endoscopic hemostasis is warranted. Previous studies have confirmed the effectiveness of several endoscopic techniques, that is, epinephrine injection, hemoclip application, and band ligation [5, 6]. However, little is known on the effectiveness of endoscopic retreatment in the MWS patients after the primary endoscopic hemostasis failure.

Combined use of hemostatic clips and detachable nylon snare (the “tulip-bundle” technique) has been described as an effective therapy for the closure of esophageal perforations after endoscopic resection [7] and of esophagomediastinal fistulas [8]. Recently, the same approach has proved to be effective as a rescue endoscopic bleeding control in the upper nonvariceal bleeding [9]. Herein, we describe the “tulip-bundle” technique as a rescue endoscopic therapy in the bleeding control in our patient with the MWS.

## 2. Case Report

An 83-year-old man with the ischaemic heart disease, gastroesophageal reflux disease, and previous peptic ulcer bleeding was admitted to our hospital with a history of haematemesis and melena. At the time of presentation, he was hemodinamically stable, and initial laboratory findings were normal. Urgent upper endoscopy revealed multiple mucosal tears above and at the gastroesophageal junction. The tear above the junction was with the active bleeding. The bleeding was arrested with combined application of epinephrine and endoclip (EZ Clip, Olympus Medical Corp, Tokyo, Japan). Further treatment included intravenous administration of fluids and proton pump inhibitors, with nihil-per-month restriction.

**Figure 1:**
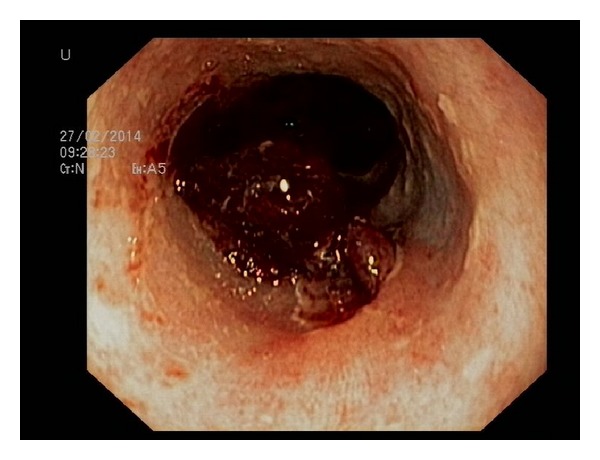
The clot in the esophagus at the site of the primary hemostasis.

**Figure 2:**
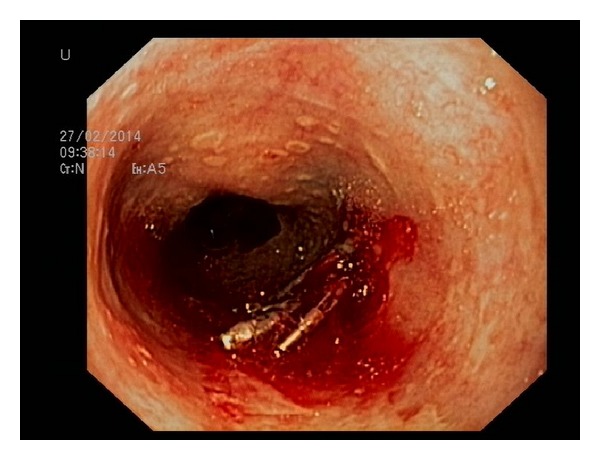
Failure of endoscopic clipping: misplacement of clips with the occurrence of bleeding.

**Figure 3:**
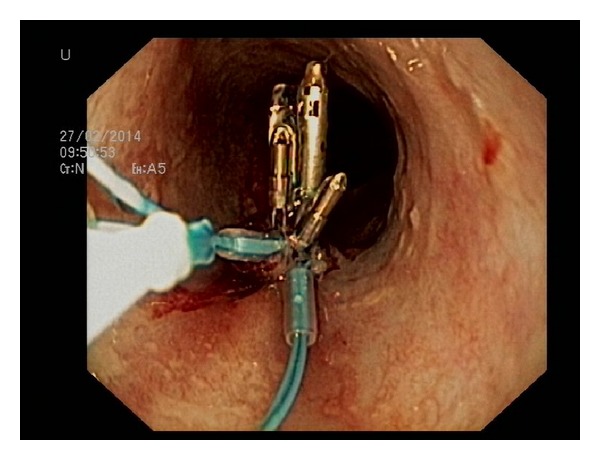
Hemostasis achieved after application of a combined use of clips and loops (“the tulip-bundle.”)

Seven hours after the procedure, the patient re-presented with retching and vomiting the fresh blood, thus prompting a second upper endoscopy. The clot in the esophagus was observed at the site of the primary hemostasis (Figure 1). After removing the clot, a mucosal tear was observed with a previously placed clip on the edge of the defect. With the intention to close the tear, two more clips (Boston Resolution Clip, Boston Scientific, Natick, Massachusets, USA) were deployed but misplaced (Figure 2) due to the constant retching of the patient during the procedure. Based on our previous experience on combined use of clips and detachable snare [10], we decided to use the same approach. Clips placed around the lesion were captured with a detachable nylon snare (Endo Loop, Olympus Medical Corp, Tokyo, Japan) and haemostasis was achieved by tightening the clips in a purse-string fashion (Figure 3). The postprocedural recovery of the patient was uneventful, and he was discharged from the hospital five days later.

## 3. Discussion

Endoscopic hemostasis with clips or thermocoagulation is the current standard in the management of the nonvariceal upper gastrointestinal bleeding [11]. Despite being very effective in achieving hemostasis, the application of clips may be difficult in some situations, depending on the location, size, and morphology of bleeding lesions. Ulcers with a fibrotic base, those located on the difficult-to-treat location (the posterior side of the duodenal bulb or the lesser curve of the stomach), or vessels with a large diameter may be less amenable to endoscopic clipping. In these circumstances, addition of another treatment modality targeting the bleeding lesion is justified as combination therapy substantially reduces the rate of rebleeding, surgery, and mortality [12].

With regard to the nonvariceal upper gastrointestinal bleeding, Lee et al. have examined the role of the tulip-bundle technique as a rescue treatment after the previous endoscopic treatment failure [9]. A total of seven patients with various gastric and duodenal bleeding lesions, in whom the primary endoscopic haemostasis was ineffective, were treated with the combination of clips and detachable snares, aiming at the endoscopic bleeding control. The technique proved to be highly efficient option for treating primary hemostatic failure with recurrent bleeding [9]. The mode of action behind the tulip-bundle technique appears to be a synergistic effect of vessel ligation and compression of the surrounding tissue defect, by virtue of a purse-string of the bundle of clips.

There were a couple of reasons behind our decision to use the same approach in our patient. This was an octogenarian, with a significant comorbidity, all of which are recognised risk factors for a fatal outcome in patients with the MWS and in whom emergency surgery would be very risky [4]. However, the over-the-scope-clip (OTSC), as a novel tool in the endoscopic armamentarium, has emerged and has been reported as an effective treatment for acute gastrointestinal bleeding after primary failure [13]; there was a fear of entrapping the previously placed clip with the “bear-claw” design of the OTSC. Finally, we had limited—but successful—experience with the combined use of clips and loop in the treatment of MWS [10].

To the best of our knowledge, this is the first case report showing the tulip-bundle technique as a rescue endoscopic bleeding control in the esophagus. Notwithstanding the fact that our case adds to the current body of knowledge in regard to effective control of nonvariceal upper gastrointestinal bleeding; the feasibility of this approach will require future studies on a large number of patients.
